# Master of Public Health programmes in South Africa: issues and challenges

**DOI:** 10.1186/s40985-017-0052-9

**Published:** 2017-02-02

**Authors:** Thembelihle Dlungwane, Anna Voce, Ruth Searle, Fred Stevens

**Affiliations:** 1 College of Health Sciences, School of Nursing and Public Health, Howard College Campus, University of KwaZulu-Natal, Glenwood, Durban, South Africa; 2 College of Health Sciences, School of Nursing and Public Health, University of KwaZulu-Natal, Durban, South Africa; 3 College of Humanities, School of Education, University of KwaZulu-Natal, Durban, South Africa; 4 Department of Educational Development and Research, Faculty of Health, Medicine and Life Sciences, Maastricht University, Maastricht, Netherlands

**Keywords:** Master of Public Health programmes, Schools of public health, South Africa, Human Resources for Health

## Abstract

**Background:**

The demand for highly skilled public health personnel in low- and middle-income countries has been recognised globally. In South Africa, the need to train more public health professionals has been acknowledged. The Human Resource for Health (HRH) Strategy for South Africa includes the establishment of public health units at district and provincial levels. Programmes such as Master of Public Health (MPH) programmes are viewed as essential contributors in equipping health practitioners with adequate public health skills to meet the demands of the health care system. All MPH programmes have been instituted independently; there is no systematic information or comparison of programmes and requirements across institutions. This study aims to establish a baseline on MPH programmes in South Africa in terms of programme characteristics, curriculum, teaching workforce and graduate output.

**Methods:**

A mixed method design was implemented. A document analysis and cross-sectional descriptive survey, comprising both quantitative and qualitative data collection, by means of questionnaires, of all MPH programmes active in 2014 was conducted. The MPH programme coordinators of the 10 active programmes were invited to participate in the study via email. Numeric data were summarized in frequency distribution tables. Non-numeric data was captured, collated into one file and thematically analysed.

**Results:**

A total of eight MPH programmes responded to the questionnaire. Most programmes are affiliated to medical schools and provide a wide range of specialisations. The MPH programmes are run by individual universities and tend to have their own quality assurance, validation and assessment procedures with minimal external scrutiny. National core competencies for MPH programmes have not been determined. All programmes are battling to provide an appropriate supply of well-trained public health professionals as a result of drop-out, low throughput and delayed time to completion.

**Conclusion:**

The MPH programmes have consistently graduated MPH candidates, although the numbers differ by institution. The increasing number of enrolments coupled by insufficient teaching personnel and low graduate output are key challenges impacting on the production of public health professionals. Collaboration amongst the MPH programmes, standardization, quality assurance and benchmarking needs considerable attention.

## Background

The rapidly changing global public health context, which includes multiple disease burdens, complex healthcare systems, and challenging socio-economic and political environments, demands corresponding changes in public health responses and competencies [1–3]. Complex public health responses demand highly skilled public health personnel, with the expertise and knowledge to contribute to strengthening health systems for optimal health service provision [2, 4–6]. However, particularly in low- and middle-income countries, huge discrepancies exist between population health needs, health service provision needs, and the quantity and appropriateness of training of the public health workforce [7, 8].

In Africa, in particular, the production of the public health workforce has not kept pace with the need, given the multiple burdens of disease, and specifically the burden imposed by the HIV, AIDS and TB epidemics [8–11]. In most African countries [2, 5, 6], health systems and health programmes are managed by clinicians with minimal public health training, resulting in poor health service management and ineffective resource allocation [2, 3]. In South Africa, the need to train more public health professionals has been acknowledged [12]. The Human Resources for Health (HRH) Strategy for South Africa includes the establishment of public health units at district and provincial levels [12]. Thus, a substantial investment is needed for training an effective health workforce.

Programmes such as the Master of Public Health (MPH) are viewed as essential contributors to equipping health practitioners to meet the demands of the health system [13, 14]. With the introduction in South Africa of a re-engineered model of primary healthcare, and a National Health Insurance (NHI), the HRH strategy has called on MPH programmes to increase the output of MPH graduates in order to ensure effective health service delivery and improved health outcomes [12].

MPH programmes in South Africa are offered mostly at postgraduate level, on a full-time or part-time basis. The programmes are aimed at equipping various health practitioners from a variety of disciplines, with key public health competencies and with collaborative strategies to address population level risk factors contributing to the global and national burden of disease [15–17]. They train health professionals by drawing on knowledge and skills from a variety of biomedical and social science disciplines to define, assess and ultimately resolve public health problems [2, 18]. In addition, the programmes equip practitioners to become innovative public health professionals with an emphasis on multidisciplinary approaches that apply the latest scientific knowledge [2, 4, 13, 18].

Postgraduate public health training in South Africa originated in medical schools in the 1940s, within departments of community medicine, and was designed as a Master of Medicine, comprising specialised public health training for medical doctors [19]. However, MPH programmes now accommodate students who come from multiple health and social science disciplines [5]. Postgraduate programmes in public health are the most broadly recognised professional postgraduate qualification for leadership positions in health [12, 20].

Most MPH programmes in South Africa are based on theoretical training with little or no field experience within the course as compared to most programmes in other African countries [21, 22]. Despite the leadership expectation of public health graduates, there are signs of a disconnection between public health leadership need and public health as taught in the MPH programmes [23]. So, the questions arise as to what is being taught in the different programmes, and what would be required to increase and upgrade the public health workforce. All MPH programmes were instituted independently. There is currently no systematic information on MPH programmes offered by South African universities. This study aims to establish a baseline on MPH programmes in South Africa (SA) in terms of programme characteristics, curriculum, teaching workforce and graduate output.

## Methods

A mixed method research design was applied. Firstly, a cross-sectional descriptive survey consisting of both quantitative and qualitative data collection was implemented of all MPH programmes offered in South Africa and active in 2014. The study did not include programmes that offer a Master of Science or Master of Philosophy in Epidemiology. The MPH programme coordinators of the 10 active programmes were invited to participate in the study via email. Out of the 10 who were invited to participate, 8 responded. Weekly reminders were sent to the non-respondents for a period of 8 weeks. An interviewer-administered questionnaire was completed with each programme coordinator between August 2014 and December 2014. The questionnaire comprised of 20 questions, both closed- and open-ended questions. Data were collected on programme characteristics, curriculum, teaching, workforce and graduate output. The questionnaire was pretested with two academic staff members in one MPH programme. The numeric data were captured into Microsoft Excel 2003 and exported into SPSS15. Numeric data were summarized in frequency distribution tables. Non-numeric data was captured, collated into one file and then thematically analysed. Throughput was determined for the cohort of students who enrolled between 2009 and 2011, and graduated between 2012 and 2014.

Secondly, document analysis was conducted of (1) curriculum documentation for each of the MPH programmes and (2) published literature on postgraduate public health training and the public health workforce in SA. Programme coordinators provided curriculum documentation. Published literature was identified through Google Scholar and PubMed (Table 1). Keywords used to search the published literature included “Master of Public Health programmes”, “Schools of public health”, “South Africa”, and “Public Health Workforce”. Given that this is a case study for SA, policy and legal documents published by South African (official) professional bodies were included. All articles and documents were written in English. The variables used were MPH programme characteristics, curriculum, teaching workforce and graduate output. The data were analysed using the four predetermined themes: context, programme structure, quality assurance processes and output.

**Table 1:** List of documents on postgraduate public health training in South Africa

Author	Year	Title
African Schools of Public Health Association	2014	Core competencies for a Master in Public Health graduate in Africa.
Gear J	2013	Public health in South Africa 1975–89: reflections on a momentous past.
Pick W	2013	Reflections on Public Health in South Africa, 1993–2002.
Hoffman M, Coetzee D, Hodes R, London L	2012	From comprehensive medicine to public health at the University of Cape Town: a 40-year journey.
Fonn S	2011	Linking public health training and health systems development in sub-Saharan Africa: Opportunities for improvement and collaboration.
National Department of Health	2011	Human Resources for Health South Africa.
Alexander L, Igumbor E, Sanders D	2009	Building capacity without disrupting health services: public health education for Africa through distance learning.
Mokwena K, Mokgatle-Nthabu M, Madiba S, Lewis H, Ntuli-Ngcobo B	2007	Training of public health workforce at the National School of Public Health: meeting Africa’s needs.
Ijsselmuiden C B NT, Duale S, Tumwesigye N M, Serwadda D	2007	Mapping Africa’s advanced public health education capacity-the AfriHealth project.
Reddy J	1998	Regional consortia, partnerships, mergers and their implications for the transformation of the South African higher education system.
National Department of Health	1997	White Paper for the Transformation of the health system in South Africa.

Ethical approval was granted by the University of KwaZulu-Natal Human and Social Sciences Research Ethics Committee (HSS/0561/014D). Informed consent was obtained from all participating MPH programme coordinators.

## Results

The results are presented in four themes, namely context, programme structure, quality assurance processes and output.

### Context

There are three types of public universities in South Africa: (a) traditional universities (*N* = 12), which offer theoretically oriented university degrees, (b) universities of technology (*N* = 8), which offer vocationally oriented diplomas and degrees, and (c) comprehensive universities, which offer a combination of both types of qualification (*N* = 6). Private universities have emerged in the last 10 years and currently, there are five accredited traditional private universities. MPH programmes are offered in eight traditional and two comprehensive public universities, whereas only one private institution offers a MPH programme (Table 2).

**Table 2:** Overview of institutions offering MPH in South Africa

Programme number	Public/private	Type of university	Learning strategy	Active/inactive	Province
Programme 1	Public	Traditional	Traditional	Active	Limpopo
Programme 2	Public	Traditional	Blended	Active	Gauteng
Programme 3	Public	Comprehensive	Traditional	Active	Limpopo
Programme 4	Public	Traditional	Traditional	Active	Gauteng
Programme 5	Public	Traditional	Traditional	Active	Gauteng
Programme 6	Public	Traditional	Blended	Active	Western Cape
Programme 7	Public	Traditional	Traditional	Active	Western Cape
Programme 8	Public	Traditional	Traditional	Active	KwaZulu-Natal
Programme 9	Public	Comprehensive	Traditional	Active	Eastern Cape
Programme 10	Public	Traditional	Traditional	Active	Eastern Cape
Programme 11	Private	Traditional	Traditional	Active	Gauteng
Programme 12	Public	Comprehensive	Online	Inactive	Gauteng

In terms of geographical location, four programmes are situated in Gauteng, two in the Western Cape, two in the Eastern Cape, two in Limpopo and one in KwaZulu-Natal.

The number of MPH programmes offered in South Africa has grown over the past 25 years, increasing from 3 in 1990 to 10 in 2014. Two universities started offering the MPH programme in 1998, followed by one in 1999, four in 2000, and one in 2006. Five MPH programmes are affiliated to medical schools and three are located within the faculty of health sciences (Table 3).

**Table 3:** MPH programme characteristics

Programme	Affiliation	Year of commencement	Stream/track	Mode of delivery	Enrolments per year	Total number of applicants per year
Programme 1	Stand-alone school	2000	Social and behavioural health, epidemiology, health system management and policy	Part time	18	120
Programme 2	Part of Medical School	2006	Social and behavioural health, epidemiology, health system management and policy	Part time	50	220
Programme 3	Stand-alone school	2000	Occupational and environmental health, health promotion, epidemiology, health administration and management; communicable and non-communicable diseases	Part time	35	140
Programme 4	Part of Medical school	1998	Health promotion, disease control, health policy and management, environmental and occupational health, monitoring and evaluation	Full time/part time	45	400
Programme 5	Part of Medical school	1998	Health systems and policy, occupational hygiene, rural health, maternal and child health, social and behavioural change communication	Part time	40	900
Programme 6	Stand-alone school	2000	General public health	Part time	40	300
Programme 7	Part of Medical school	1999	General public health, health systems, health economics, social and behavioural sciences, epidemiology and biostatistics	Part time	65	
Programme 8	Part of Medical school	2000	General public health	Part time	65	150

### Programme structure

The number of enrolments per year ranges from 20 to 80 across MPH programmes. All programmes have increased enrolment numbers, in line with the government mandate of the massification of, and increased access to higher education [24]. In the years 2012 to 2014, the number of new applications received by individual MPH programmes per year outnumbered the number of vacancies (Table 3). Applicants are not only from South Africa but from all over Africa and further afield. In order to qualify to enter MPH studies, an applicant must possess at least a four-year bachelor level degree in the social sciences or in the health sciences and, in some instances, 2–3 years of work experience is a prerequisite.

Most of the programmes offer intensive face-to-face lectures at the beginning of the semester. In addition, course material and assignments are provided through online teaching platforms. Most of the programmes are university based with little or no service attachment or field placement. The total number of full-time academic staff across all MPH programmes was 84 (Table 4). The student: staff ratio ranged from 1:7.25 to 1:24.25 across programmes. The number of academic staff with PhDs is low in some programmes (Table 4).

**Table 4:** Total number of full time academic staff and educational level

Institution	No of academic staff	Educational level	
		Masters	PhD
Programme 1	4	3	1
Programme 2	12	5	7
Programme 3	7	3	4
Programme 4	20	4	16
Programme 5	4	1	3
Programme 6	22	11	11
Programme 7	15	5	10
Programme8	10	7	3
Total	94	39	55

Most programmes are offered part time. The delivery of the programmes caters for students who are enrolled for postgraduate studies while working. Two programmes are offered via distance education with only initial face-to-face contact during the first semester in the first year. A majority of programmes offers a wide range of specialisations. All programmes comprise both a coursework and a research component. The weighting of coursework compared to research credits varies from institution to institution. However, the coursework and research components have to meet the minimum proportion of credits according to the National Qualifications Framework (NQF).

With regard to the coursework component, the programmes offer a general MPH track as well as specialisation tracks. The common core modules for the general MPH track are biostatistics and epidemiology. The most common specialisation tracks across programmes are epidemiology, social and behavioural sciences, and health policy and management (Table 2). For the research component, most institutions expect students to complete a mini thesis that comprises at least one third (33%), and up to one half (50%), of the total qualification credits (Table 5).

**Table 5:** MPH curricula in South Africa

Programme	Credits required	Coursework	Research project	Core modules	Module offering	Total number of modules required
Programme 1	200	60%	40%	6	8	8
Programme 2	200	50%	50%	4	8	8
Programme 3	180	67%	33%	3	8	8
Programme 4	240	67%	33%	10	51	10
Programme 5	360	50%	50%	7	12	6
Programme 6	180	67%	33%	6	8	8
Programme 7	180	67%	33%	4	25	10
Programme 8	192	50%	50%	5	9	6

In SA, no consensus has been reached with regard to core competency guidelines for MPH graduates and to date, no universal core competencies have been adopted. [25]. Hence, the curriculum varies from programme to programme. The development of a competency framework was drafted by the African Association of Schools of Public Health in 2014. The current draft was tabled for discussion by relevant stakeholders. The draft outlines that MPH competencies should address the current state of health systems and burden of disease in Africa which is characterised by epidemics of both communicable and non-communicable diseases [25].

### Quality assurance processes

All Master’s programmes in South Africa are guided by the NQF in terms of the minimum number of credits required for the awarding of a master level qualification. The current MPH programmes are run by individual universities, each having their own quality assurance, validation and assessment processes with minimal external scrutiny. Some programme coordinators highlighted the need for an association or quality assurance body to ensure that there is benchmarking and standardization across various programmes. A proposal to establish standards-generating bodies for all programmes was released in 2000, but there has never been one established for public health postgraduate programmes [26].

### Outputs

**Fig. 1:**
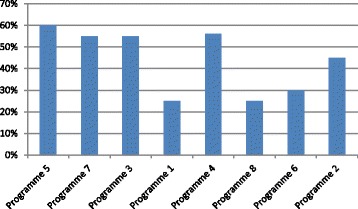
Throughput of MPH programmes: students enrolled between 2009 and 2011 and graduated between 2012 and 2014

Over the past 5 years the total number of graduates has steadily increased (Table 6). However, the yearly average throughput for MPH programmes in South Africa ranged from 25 to 60% for the cohort of students who enrolled between 2009 and 2011, and graduated between 2012 and 2014 (Fig. 1). The peak attrition period in most MPH programmes is on completion of the coursework prior to engaging in the research project.

**Table 6:** MPH graduates: 2010–2014

Institution	2010	2011	2012	2013	2014
Programme 1	–	–	–	–	–
Programme 2	27	35	11	24	43
Programme 3	10	6	5	13	4
Programme 4	43	38	32	36	29
Programme 5	14	39	31	19	23
Programme 6	38	42	54	29	26
Programme 7	23	36	32	44	20
Programme 8	11	11	4	12	11

The average time to completion amongst MPH students across all programmes is 4–5 years, as opposed to the minimum 2 years for part-time studies recommended by the South African Qualifications Authority (SAQA). Drop-out and delays are attributed to the research project challenges experienced by students. Challenges experienced in the research project identified by programme coordinators include inadequate academic writing skills among students and poor supervisor-student relationships.

## Discussion

There are limited number of MPH training institutions in SA. Of the 20 traditional and comprehensive public universities, the MPH is only offered in 10 institutions across the country. The training is concentrated in few provinces. For a population of 56 million in SA, public health training is not widely distributed and public health capacity is concentrated in a few urban-based academic institutions. However, this is not unique to SA. Several low- and middle-income countries (LMICs) are faced with inadequate numbers of educational institutions and geographical maldistribution of MPH training programmes [6, 7, 27–30].

Postgraduate public health training in SA dates back to the 1940s, where multidisciplinary training was offered by the Institute for Family and Community Health (IFCH) [31, 32]. The institute was later closed due to the lack of political support [31, 32]. In most medical schools, postgraduate public health was offered for medical doctors only, within departments of community medicine, which later evolved into departments of community health [6, 33].

The expansion of postgraduate public health training in SA developed in parallel with the advancement of the health system, fuelled by profound political changes that took place in SA in 1990s [34, 35]. The transition of SA to democracy in 1994 brought about health sector reforms and a new approach to health planning and health systems management. In 1997, a white paper for the transformation of the health system was promulgated, which called for the establishment of the district health system based on a primary health care approach [36]. The white paper also emphasized the need for a reorientation of health professional training, advocating for a strong focus in training on population-based health planning, health promotion and education, and on the development of foundational public health functions [36]. In response to the call made by the white paper for the transformation of the health system, postgraduate public health training was then introduced. This led to the initial formation of three regional schools of public health offering MPH programmes, Gauteng (previously Transvaal), KwaZulu-Natal and Western Cape [34, 35].

The South African public health sector landscape has changed over the years and is in the process of implementing a National Health Insurance (NHI) and a re-engineering of primary health care. These initiatives require adequate human resources to support the health system at all levels [20]. Even though the HRH strategy highlights the need to upscale the number of public health professionals, the South African health system remains a curatively oriented health service [37, 38]. The allocation of resources focuses more on clinical services, with minimal attention given to health promotion and prevention [37, 38].

South Africa’s public health history is rooted in fragmented and weak health system performance [9, 38]. This is further exacerbated by the changing epidemiological transitions characterised by persistent infectious disease and emerging non-communicable disease burdens [9, 38, 39]. Effective leadership and stewardship is critical in improving the performance of the entire health system [3, 40]. The existing undersupply of public health professionals and shortfalls in the managerial and supervisory capacity is a threat to the success of a NHI scheme [12, 20]. MPH programmes are viewed as potential contributors to address the current health system leadership deficiencies [13, 33, 41, 42]. However, the programmes might not be able to meet the need due to the lack of hands-on field practice within the training programmes. Factors contributing to the disconnection between public health leadership needs and what is taught may require further investigation.

Despite the rise in the number of public health programmes, the demand for public health postgraduate education seems to exceed what current MPH programmes can supply, both in terms of workplace demands for health professionals with public health competencies and in terms of rising student enrolments. One of the issues emerging for several MPH programmes is the inadequate number of academics, which puts an extra burden on existing staff. In addition, the number of staff with doctorates was inadequate for some MPH programmes which influences the “scholarship” in public health and public health training. The South African government is making efforts to enhance the qualifications of the academic staff, with the emphasis on doctoral training [43]. The paucity of academics with PhDs poses a challenge, given that the quality of higher education is not only determined by the number of staff but by their qualifications and research experience [44]. The staff shortages and scarcity of academics with PhDs have been reported in resource-poor countries and are largely attributable to the “brain drain” and small student enrolment numbers [6, 28, 43–45].

Public health competencies have been under scrutiny in a number of countries across the globe. In the USA, core public health competencies were established by the Council on Linkages between Academia and Public Health Practice in 2010. The Association of Schools in Public Health drafted a list in 2008, which was redefined in 2011 [46, 47]. The United Kingdom Public Health Skills and Career Framework was endorsed in 2008 [47, 48], whereas in Australia, the Foundation Competencies for MPH alumni were published in 2009 [49]. Professional competencies need to be context specific [47, 50–52]. MPH graduates should possess and demonstrate skills and competencies in leadership, systems thinking, policy development, critical and analytical thinking, and teamwork and communication skills in order to improve health system performance and population health [30, 47, 52, 53].

Programmes need to identify core competencies for each curriculum domain [50, 51]. Core competencies for MPH programmes in SA have not been concluded. The current draft, under review by relevant stakeholders, outlines that MPH competencies should take into account the African health context which is characterised by epidemics of both communicable and non-communicable diseases [25].

The ailing health system and poor leadership and stewardship in the health sector, coupled with high burden of disease and emerging epidemics requires adequate and highly trained public health professionals [3, 34, 54]. The MPH programmes contribute to addressing competencies such as context-specific leadership, planning, and management [47]. Therefore MPH programmes in Africa should include competencies on leadership and health system strengthening [25].

The absence of an overarching body responsible for oversight of in SA ensuring benchmarking and standardization across programmes, is a challenge. There are countries that have a single independent council that covers conventional public health training for MPH programmes [37, 38]. Replicable models from those countries might assist in ensuring quality in public health training [55, 56]. According to Fonn, a close partnership between the MPH programmes and national health systems must be established in order to ensure that public health training informs health systems planning and management [3].

Public health training is crucial in order to address the current discrepancies in HRH. However, the low throughput and delayed time to completion experienced by MPH programmes negatively affects the production of highly skilled practitioners for the health sector. The recommended 2 years for completing part time Master’s programme is not always feasible for South African MPH students who are mostly mature students, in full time employment, and have other responsibilities [27, 57]. Therefore, MPH programmes need to devise strategies to assist students, especially when it comes to dissertation writing, which is reportedly when most students tend to delay or drop-out. Strategies could include cohort writing seminars and provide mentorship through alumni network and faculty staff as an on-going process for MPH students [57–59]. In addition, further research needs to be conducted to investigate factors contributing to student drop-out in MPH programmes.

### Limitations

MPH programme coordinators that were interviewed all came from public universities who have offered the programme for more than 10 years. The two MPH programmes that did not participate are fairly new. There is one private institution that offers MPH training in South Africa. The perspectives of programme coordinators within the private institution may differ from programme coordinators that responded.

Based on the findings of this study and the literature cited, further research needs to be conducted on the development of a competency framework for MPH programmes in South Africa. Further research should focus on graduates’ and employers’ perspectives on the skills and competencies acquired from MPH programmes and how these impact the health sector. Furthermore, future studies will need to investigate the characteristics and career pathways of MPH graduates. Moreover MPH outputs and job match needs to be assessed.

## Conclusions

MPH programmes are contributing to the public health training of the health workforce in South Africa. The increasing number of enrolments coupled by insufficient teaching personnel and low graduate output are key challenges impacting on the adequate production of health professionals with public health competencies. The absence of collaboration amongst the MPH programmes and the lack of standardization, quality assurance and benchmarking need considerable attention. Moreover, effective governance and investment in management development and research development (PhD graduates) are essential to strengthen the capacity of MPH programmes. This may result in adequate training of public health practitioners and overcome the current HRH constraints.

## Abbreviations

HIV/AIDS: Human immunodeficiency virus/acquired immune deficiency syndrome
HRH: Human Resource for Health
MPH: Master of Public Health
NHI: National Health Insurance
NQF: National qualification framework
SA: South Africa
